# Supplementary material for: Evaluation of potential reference genes for quantitative RT-PCR analysis in spotted sea bass (Lateolabrax maculatus) under normal and salinity stress conditions
Source: PeerJ. 2018 Sep 19;6:e5631. doi: 10.7717/peerj.5631 (PMC6151123; doi:10.7717/peerj.5631)
Supplement: Figure S1 — Dissociation curves of 9 candidate reference genes 18S rRNA (A); ACTB (B); B2M (C); EF1A (D); GAPDH (E); HRPT (F); RNAPol II (G); RPL7 (H); TUBA (I), reveal single peaks, which obtained from three technical replicates of different cDNA samples. [file peerj-06-5631-s001.docx]

Figure S1. Specificity detection of primers for each candidate genes by melting curves.


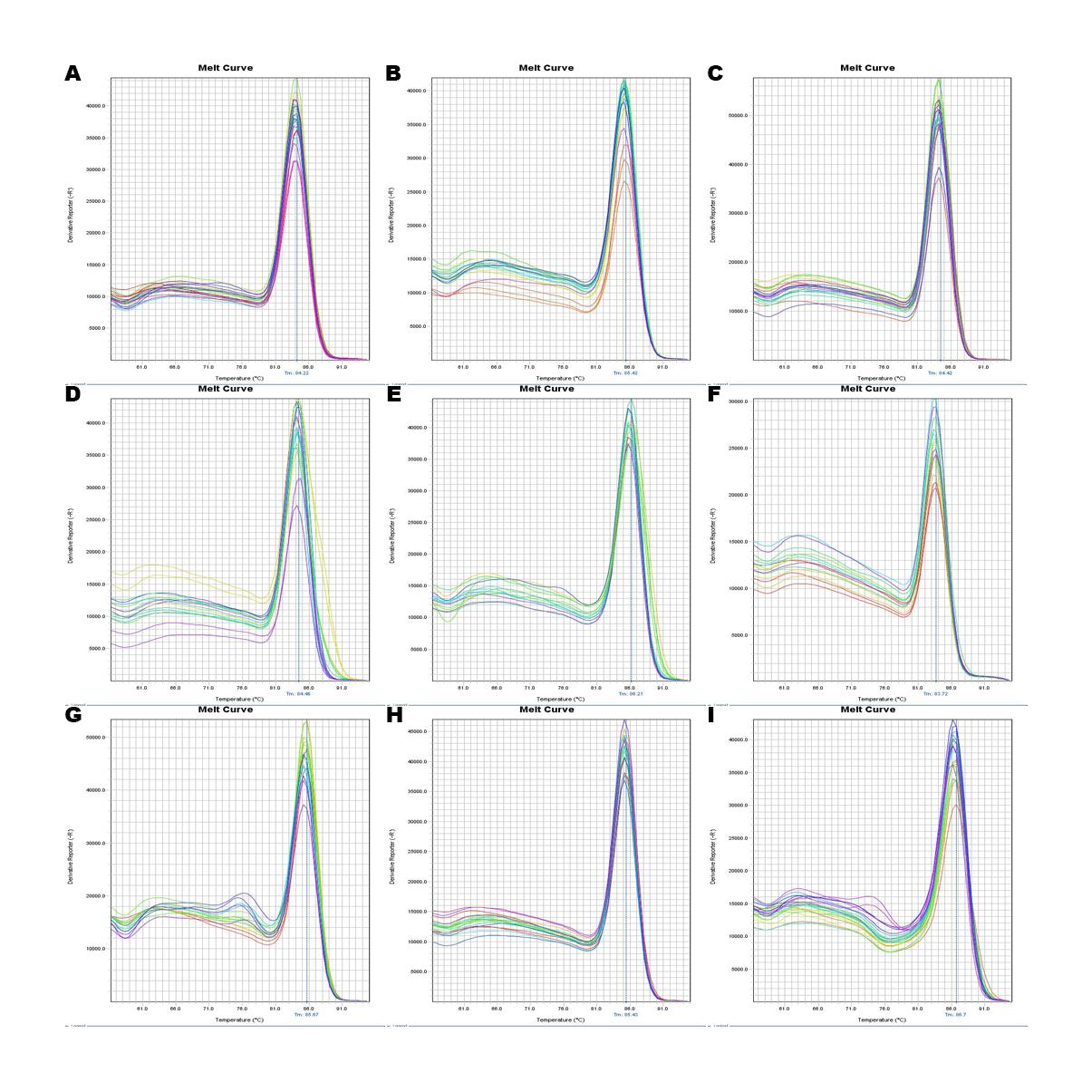


Dissociation curves of 9 candidate reference genes *18S rRNA* (A); *ACTB* (B); *B2M* (C); *EF1A* (D); *GAPDH* (E); *HRPT* (F); *RNAPol II* (G); *RPL7* (H); *TUBA* (I), reveal single peaks, which obtained from three technical replicates of different cDNA samples.
