# Supplementary material for: Evaluation of potential reference genes for quantitative RT-PCR analysis in spotted sea bass (Lateolabrax maculatus) under normal and salinity stress conditions
Source: PeerJ. 2018 Sep 19;6:e5631. doi: 10.7717/peerj.5631 (PMC6151123; doi:10.7717/peerj.5631)
Supplement: Figure S2 — Trans2K DNA marker (A); RNAPol II (B); RPL7 (C); HRPT (D); GAPDH (E); TUBA (F); B2M (G); 18S rRNA (H); EF1A (I); ACTB (J). [file peerj-06-5631-s002.docx]

Figure S2. 1.5 % agarose gel electrophoresis is exhibiting specific qRT-PCR products with expected size for each candidate reference gene.


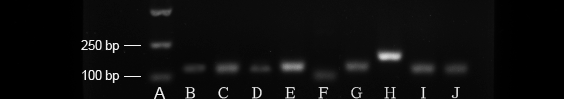


Trans2K DNA marker (A); *RNAPol II* (B); *RPL7* (C); *HRPT* (D); *GAPDH* (E); *TUBA* (F); *B2M* (G); *18S rRNA* (H); *EF1A* (I); *ACTB* (J).
