# Supplementary material for: Evaluation of potential reference genes for quantitative RT-PCR analysis in spotted sea bass (Lateolabrax maculatus) under normal and salinity stress conditions
Source: PeerJ. 2018 Sep 19;6:e5631. doi: 10.7717/peerj.5631 (PMC6151123; doi:10.7717/peerj.5631)
Supplement: Table S1 [file peerj-06-5631-s003.docx]

Table S1. NormFinder analysis estimates of intra and intergroup variations.

Intragroup variation in different tissues.

| Group identifier/  Gene | intestine | muscle | spleen | stomach | brain | heart | liver | gill | kidney | pectoral fins |
| --- | --- | --- | --- | --- | --- | --- | --- | --- | --- | --- |
| *ACTB* | 0.165 | 0.061 | 0.107 | 0.002 | 0.029 | 0.227 | 0.197 | 0.182 | 0.293 | 1.794 |
| *18S rRNA* | 0.408 | 0.002 | 0.057 | 0.020 | 0.035 | 0.000 | 0.001 | 0.003 | 0.000 | 2.042 |
| *B2M* | 0.019 | 0.004 | 0.166 | 1.297 | 0.000 | 0.000 | 0.001 | 0.073 | 0.039 | 0.000 |
| *EF1A* | 0.000 | 0.000 | 0.296 | 0.007 | 0.011 | 0.020 | 0.162 | 0.000 | 0.000 | 1.453 |
| *GAPDH* | 0.041 | 0.028 | 0.109 | 0.014 | 0.127 | 0.000 | 0.001 | 0.064 | 0.371 | 0.001 |
| *HRPT* | 0.000 | 0.004 | 0.000 | 0.002 | 0.038 | 0.471 | 0.477 | 0.033 | 0.003 | 1.222 |
| *RNAPol II* | 0.118 | 0.002 | 0.004 | 0.029 | 0.034 | 0.116 | 0.001 | 0.000 | 0.021 | 0.001 |
| *RPL7* | 0.000 | 0.002 | 0.030 | 0.102 | 0.285 | 0.207 | 0.425 | 0.196 | 0.022 | 0.000 |
| *TUBA* | 0.086 | 0.021 | 0.096 | 0.240 | 0.000 | 0.271 | 0.141 | 0.375 | 0.281 | 4.367 |

Intergroup variation in different tissues.

| Group identifier/  Gene | intestine | muscle | spleen | stomach | brain | heart | liver | gill | kidney | pectoral fins |
| --- | --- | --- | --- | --- | --- | --- | --- | --- | --- | --- |
| *ACTB* | -1.076 | 0.113 | -0.274 | 0.624 | 0.338 | -0.093 | -0.579 | 0.467 | -0.341 | 0.821 |
| *18S rRNA* | -1.289 | 0.281 | 0.196 | 0.173 | 0.249 | -0.241 | -1.027 | 0.599 | -0.048 | 1.107 |
| *B2M* | 0.210 | -2.281 | 1.560 | 0.301 | -1.026 | 0.258 | -0.654 | 1.944 | -1.486 | 1.173 |
| *EF1A* | -0.069 | -1.935 | 0.437 | -0.261 | -0.748 | 0.126 | 0.831 | 0.375 | -0.060 | 1.304 |
| *GAPDH* | 1.790 | 6.488 | -3.555 | 1.563 | -1.337 | 1.399 | 1.069 | -4.836 | 1.748 | -4.329 |
| *HRPT* | -0.183 | -0.261 | 0.986 | -0.765 | 0.369 | 1.050 | 0.359 | -0.565 | 0.455 | -1.445 |
| *RNAPol II* | 0.357 | -1.548 | 0.305 | -0.980 | 0.743 | -0.490 | -0.125 | 0.673 | 0.050 | 1.015 |
| *RPL7* | 0.425 | -0.638 | 0.310 | -0.506 | -0.643 | -1.059 | 0.428 | 1.045 | -0.864 | 1.502 |
| *TUBA* | -0.165 | -0.220 | 0.035 | -0.149 | 2.057 | -0.949 | -0.303 | 0.297 | 0.546 | -1.148 |

Best combination of two genes is *ACTB* and *HRPT.*

Intragroup variation in salinity stress.

| Group identifier/  Gene | 0 ppt | 12 ppt | 30 ppt | 45 ppt |
| --- | --- | --- | --- | --- |
| *18S rRNA* | 0.051 | 0.145 | 0.009 | 0.080 |
| *ACTB* | 0.002 | 0.002 | 0.001 | 0.046 |
| *B2M* | 0.003 | 0.000 | 0.013 | 0.018 |
| *TUBA* | 0.002 | 0.001 | 0.007 | 0.000 |
| *GAPDH* | 0.000 | 0.259 | 0.035 | 0.011 |
| *HRPT* | 0.001 | 0.008 | 0.011 | 0.008 |
| *RNAPol II* | 0.016 | 0.000 | 0.000 | 0.000 |
| *EF1A* | 0.016 | 0.000 | 0.009 | 0.079 |
| *RPL7* | 0.015 | 0.021 | 0.000 | 0.000 |

Intergroup variation in salinity stress.

| Group identifier/  Gene | 0 ppt | 12 ppt | 30 ppt | 45 ppt |
| --- | --- | --- | --- | --- |
| *18S rRNA* | 0.005 | -0.102 | -0.320 | 0.418 |
| *ACTB* | -0.401 | 0.355 | 0.027 | 0.019 |
| *B2M* | 0.221 | 0.252 | -0.084 | -0.389 |
| *TUBA* | 0.010 | 0.456 | -0.200 | -0.267 |
| *GAPDH* | 0.004 | -1.125 | 0.715 | 0.406 |
| *HRPT* | 0.030 | 0.209 | 0.102 | -0.342 |
| *RNAPol II* | 0.132 | -0.248 | -0.154 | 0.269 |
| *EF1A* | 0.079 | 0.264 | -0.032 | -0.311 |
| *RPL7* | -0.081 | -0.062 | -0.054 | 0.196 |

Best combination of two genes is *HRPT* and *RNAPol II.*
