# Supplementary material for: Evaluation of potential reference genes for quantitative RT-PCR analysis in spotted sea bass (Lateolabrax maculatus) under normal and salinity stress conditions
Source: PeerJ. 2018 Sep 19;6:e5631. doi: 10.7717/peerj.5631 (PMC6151123; doi:10.7717/peerj.5631)
Supplement: Table S2 — The sequence information of these 9 reference genes including B2M, GAPDH, TUBA, EF1A, HRPT, RNAPol II, RPL7, ACTB, 18SrRNA. [file peerj-06-5631-s004.docx]

**The sequence information of these 9 reference genes including *B2M*, *GAPDH*, *TUBA*, *EF1A*, *HRPT*, *RNAPol II*, *RPL7*, *ACTB*, *18SrRNA***

>Seq1 [organism=Lateolabrax maculatus ] Lateolabrax maculatus (spotted sea bass) β-2-microglobulin (B2M) mRNA, complete cds

AAGATCCATCAAGCAACATTTTTCATATGAATATCAAATTATATGATGGCTCCCTATAATGGTGAAGTTTTCAGCTACTTTGTTTTGAGGGCAGCCACTCTCACCTCACAGCTGACTGGACACTTCCACAGGGAGGAACGGAGCCAGGAGCCATCGTGTCTGACAAAAATCAATCATCCGTGCCAACTAAAAACTGAATATTTTTTTCAATGCCCTAATTAGTTTTGATAAATTAAATGTAATTCATTCACCCATTGTTAAAATGACACATTTTTAGGAACACAGTCTGATGATGTGAACACAAGCTTGGATGTGTTAAATTAACTGGAGACAGTGTGGTGGAATCTGCCTTACTGTCATTTTTTTCTAGTAGCAAACTGTGTTGCTACAACAGAGGAAACTACTGATGTTTCCTCATAGACTGCAATTATCAAACATACATCTTATAAACACCACAGATGGCAGAAGGTAAATTTAAAACTATAGACAATACATTATGATCTTCTGAGCTTGAGACCAAGCAGACCTGAAGCAAGGCGGAGCGCAGAACTTACATATCTGGTTCCCAAGTGTACATTTTGGACGTTCCCCCGTGAGTCACCCTGCAGGCGTAGTCGTCTCCTTTGACTGGGGTGAAGGTCACGTGTCTGGTCAGGTGGTAGTGCCAGTCCTCCTCGAAGGCCAGGTCGGTCTGATTGGCTTCAGGTATTAACTCTCCATTCCTGAGCAGCTCAATGGTGATTTCTGGCGGGTAAAAGCCGCTGGCATGACAGATGAAGGTGTTGGGCTTGTCGAACACTCCCGGGCCATAGCTGTACACCTGAACCTTGGGTGCCAATTCTTTAGCGATTGAAAGCGCCAGGAGGCAGAGCAGACCGGCCACAACTGCGCACACAAATCCCCTCATGGCTTCACCTTCAGTCGCTCTCTGTACGCTGGGAAGGCTGAAGTTATTGTATGTTTGTCCTAACAAGAAGGACGACGAAATGGCTCCGCGATCCAAAACGAAAGTAAAAAAAAA

>Seq2 [organism=Lateolabrax maculatus ] Lateolabrax maculatus (spotted sea bass) Glyceraldehyde-3-phosphate dehydrogenase (GAPDH) mRNA, complete cds

CCCCACTCACACCCGGAGTCGCAAGACAGACTGAGGAGTCTCACAAACGAGGACTAAGAACAAGCCAGGCAAAATGGTGAAAGTCGGTATCAATGGATTCGGCCGCATCGGTCGTCTGGTGACCCGTGCTGCCTTCACCTCCAAGAAGGTGGAGATTGTGGCCATCAATGACCCTTTCATCGACCTGGAGTACATGGTCTACATGTTCAAATATGACTCCACCCACGGCCGCTTCAAGGGTGAGGTCAAGATTGAGGGTGACAAGCTGGTCATCGATGGACATAAAATCAGCGTTTTCCATGAGAGAGACCCCACTAACATCAAATGGGGGGATGCTGGTGCCCAGTACGTGGTTGAGTCCACTGGTGTGTTCACCACCATCGAGAAGGCCTCTGCTCACTTGAAGGGGGGTGCCAAGAGAGTCATCATCTCTGCACCCAGCGCTGATGCTCCCATGTTTGTCATGGGTGTCAACCATGAGAAGTACGACAAATCCCTCCCGGTTGTCAGCAATGCTTCCTGCACAACCAACTGCCTGGCCCCTCTGGCCAAGGTCATCAATGACAACTTCGGCATCATTGAGGGCCTGATGAGCACAGTCCATGCCATCACTGCCACCCAGAAGACTGTGGACGGTCCCTCCGGCAAGCTGTGGAGGGACGGCCGTGGTGCCAGCCAGAACATCATCCCCGCTTCTACTGGTGCTGCCAAAGCTGTCGGCAAGGTCATCCCTGAGCTCAATGGCAAGCTGACTGGCATGGCCTTCCGTGTCCCCACCCCCAACGTGTCAGTGGTTGACCTGACAGTCCGTCTGGAGAAACCCGCCAAATACGAGGACATCAAGAAGGTTGTGAAGGCCGCAGCTGATGGACCCATGAAGGGCATTCTGGGATACACAGAGCACCAGGTCGTCTCCACAGACTTCAATGGTGACACTCACTCCTCCATCTTTGATGCTGGCGCTGGCATCGCCCTTAATGATCACTTTGTCAAGCTGGTCACATGGTACGACAATGAGTTCGCATACAGCAACCGTGTCTGCGACCTGATGGCCCACATGGCTACCAAGGAGTAAACCAACCAGCCAATCGCCCGTCACTGCACTATGCCGCATCTACCACATTCTCCTAATACCAGAGTTATAAATGTACAATAAGACATTGTATTATCTATGCCTGGTCCCATCAGATAGTAGACCACCATTAGATGCCTGAGGGAAGATCCCACGGAGAGCAAACGAAAAAAGATTTAAATTGGTGACCAATTACTTTTTTTTATTTGTTAAATTCCCTGCTCTGTTCTTGTGTAAGATGGAATGAAGGTGTTAGAGTCTTTGTCTGTCTGTGGTACTGAAGTACTGCAGTCAGTGGTGGAATAAAGTCCTCTGTTT

>Seq3 [organism=Lateolabrax maculatus ] Lateolabrax maculatus (spotted sea bass) α-Tubulin chain(TUBA) mRNA, complete cds

CTTAGTCTCTTATTCTTCTTAATTCAAACCTCCACCGACTCCACACCTTCAGGAATCATGCGTGAATGTATCTCAGTGCACGTTGGTCAGGCCGGTGTCCAGATTGGCAATTCCTGCTGGGAGCTTTACTGCCTGGAGCATGGGATCCAGCCGGACGGACAGATGCCCAGTGGCAAGACCATCGGGGGAGGAGACGATTCCTTCAATACCTTCTTCAGTGAGACTGGAGCCGGAAAGCACGTCCCCAGGGCTGTCTTTGTGGACCTGGAGCCCACCGTCATCGATGAGGTGCGCTCTGGGACCTACCGCCAGCTGTTCCACCCTGAGCAGCTGATCACAGGCAAGGAAGATGCTGCCAACAACTACGCCCGTGGACACTACACCATCGGCAAGGAAATCATTGATCTGGTTTTGGACAGGACTCGCAAACTGGCTGATCAGTGCACTGGCCTGCAAGGATTCCTCATCTTCCACAGCTTTGGTGGAGGCACTGGTTCAGGTTTCACCTCCCTGCTGATGGAAAGACTCTCTGTTGATTATGGAAAGAAATCAAAACTTGAATTTGCCGTCTACCCAGCCCCACAGGTCTCCACAGCAGTAGTAGAGCCTTACAACTCCATCCTAACCACCCACACCACCTTGGAGCACTCCGACTGTGCCTTCATGGTGGACAATGAGGCCATCTACGACATCTGC

>Seq4 [organism=Lateolabrax maculatus ] Lateolabrax maculatus (spotted sea bass) Elongation factor-1-α (EF1A) mRNA, partial cds

TTTCCCATTGTTTTTAGTTTCGGCTCTTTCTGTTACCTGGCGAGGGGCAGCAGCTTTGGCCGTGAAAACCCAGAAACACCGAAACTAAAAACAATGGGAAAGGAAAAGATCCACATCAACATCGTGGTCATTGGCCATGTCGACTCCGGCAAGTCCACCTCCACCGGCCACTTGATCTACAAGTGCGGAGGAATCGACAAGAGAACCATCGAGAAGTTCGAGAAGGAAGCCGCTGAGATGGGCAAGGGCTCCTTCAAGTACGCCTGGGTGCTGGACAAACTGAAGGCCGAGCGTGAGCGTGGTATCACCATTGACATCGCTCTGTGGAAGTTTGAGACCGGCAGGTACTACGTCACCATCATTGATGCCCCTGGACACAGGGACTTCATCAAGAACATGATCACTGGAACCTCTCAGGCTGACTGCGCTGTGCTGATTGTTGCTGCTGGTGTTGGTGAGTTCGAGGCTGGTATTTCCAAGAACGGCCAGACCCGTGAGCACGCTCTGCTCGCCTTCACCCTGGGTGTGAAGCAGCTCATCATTGGTGTCAACAAGATGGACTCCACCGAGCCCCCTTACAGCCAGTCCCGCTTTGAGGAAATTTCCAAGGAAGTGAGCACCTACATCAAGAAGATCGGCTACAACCCCGCCGCTGTTGCCTTTGTCCCCATCTCTGGATGGCACGGAGACAACATGCTGGAGCCCAGTGAGAAGATGAGTTGGTACAAGGGATGGAAGATTGAGCGCAAGGAGGGCAATGCTAGCGGAACCACACTGCTGGAGGCTCTTGATGCCATCCAGCCTCCCAGCCGCCCCACAGAGAAGCCCCTCCGTCTGCCCCTGCAGGACGTCTACAAAATCGGCGGTATTGGAACTGTACCTGTCGGTCGTGTGGAGACTGGTATCCTGAAGCCCGGTATGGTTGTCACCTTCGCTCCCGCCAACCTGACCACTGAGGTGAAGTCTGTGGAGATGCACCACGAGACTCTGCCCGAGGCTGTCCCTGGTGACAATGTTGGCTTCAACATCAAGAACGTGTCCGTCAAGGAAATCCGTCGTGGATTCGTTGCTGGAGACAGCAAGAACGACCCTCCCAAGGGAGCTGACAACTTCAACGCCCAGGTCATCATCCTGAACCACCCCGGCCAGATCTCCCAGGGTTACGCCCCTGTGCTGGATTGCCACACCGCTCACATTGCCTGCAAGTTCAGGGAGCTCATCGAGAAGATCGACCGTCGTTCTGGCAAGAAGATTGAGGACGCACCCAAGTTTGTCAAGTCTGGAGACGCCGCCATTGTTAACCTGGTTCCACAGAAGCCAATGGTTGTGGAGCCTTTCTCCAACTACCCTCCCCTCGGTCGTTTTGCTGTGCGTGACATGAGGCAGACGGTGGCTGTCGGTGTCATCAAGGCCGTTGAGACCAAGGAAGTATCCGGAAAGACAACCAAGGCTGCAGAGAAGGCCCAGAAGAAGAAATGA

>Seq5 [organism=Lateolabrax maculatus ] Lateolabrax maculatus (spotted sea bass) Hypoxanthine guanine phosphoribosyl transferase1 (HRPT) mRNA, partial cds

AGAGTCCGCGGCAACAGCTCTGACGACACGTCCAGCGTCTCCTCAAACCGAAGACGGCGGCAGACCACGGAAAACGGAATGGCGGGGACCGACGCGGACATGGCGACACCCAACCCCTGTGTTGTGATCAGTGATGAAGAGCAGGGGTATGACCTGGACCTTTTCTGCATACCAAAACATTATGCCACTGACCTGGAGAGGGTCTACATCCCACATGGACTCATCTTGGACAGGACAGAGAGACTGGCCAGAGAGATCATGAAGGAAATGGGGGGGCACCACATCGTGGCCCTCTGTGTGCTCAAAGGGGGTTACAAGTTCTTTGCTGACCTGCTGGACTACATCAAGGCCCTGAACAGGAACAGTGACCGCTCCATTCCAATGACAGTGGACTTCATCCGCCTCAAGAGCTACTGTAACGACCAGTCGACAGGGGAAATCAAAGTAATTGGAGGAGATGACCTGTCTACATTGACAGGCAAGAATGTCTTGATTGTGGAGGATATTATCGACACAGGGAAGACAATGAAGACTTTATTG

CAACTCCTCAAACAGTACAATCCCAAAATGGTTAAAGTAGCAAGTTTGTTGGTGAAGAGAACACCAAGAAGTGTTGGCTACCGACCGGACTTTGTAGGATTCGAGGTCCCTGACAAATTTGTGGTGGGATACGCACTAGACTACAACGAGTACTTTAGAGATCTAAATCATATCTGCGTCATAAGTGAAACAGGGAAGGAGAAGTACAAGGCATGA

>Seq6 [organism=Lateolabrax maculatus ] Lateolabrax maculatus (spotted sea bass) RNA polymerase II subnuit C(RNAPol II) mRNA, complete cds

AAGGCAGAAACAGCGGCAGTGGAGTGTGTGGCCGACTTTACGACGTTAACTTTGGGCTTTTTTAACTCCAATAATGCCGTATGCTAACCAACCCACCGTGAAGATCACAGAACTGACAGACGAAAATGTCAAGTTCGTCATTGAAAACACTGATTTGGCGGTTGCAAACTCCATCCGCCGCGTCTTCATGTCAGAAGTTGCCACTATAGCCATTGACTGGATCCAGATCGATGCCAATTCGTCGGTGCTGCATGATGAGTTTGTTGCACACAGAGTTGGTCTCATACCTCTCACAAGCGACGACATCGTGGACAAAATGCAGTATTCTAGGGACTGTACCTGTGATGATTTTTGTCCGGAGTGCTCTGTTGAGTTGACGCTGGACGTTCGATGTACTGAGGACCAGACACGTCATGTAACCTCACGTGACCTTTTGTCCAACAACCCACGAGTCATCCCGGTGACTTCCAGGAGTCGAGACAATGATCCCAATGACTACGTTGAACAAGATGACATTTTACTGGTGAAGCTTCGTAAAGGTCAGGAACTACGGCTCAGGGCGTACGCCAAGAAGGGCTTTGGTAAGGAGCACGCCAAGTGGAACCCAACAGCAGGGGTTTCCTTCGAGTACGACCCAGACAATGCACTGAGGCACACAGTCTACCCACGACCGGAGGAGTGGCCAAAGAGTGAATACTCAGAGATTGAGGAGGATGAGGTTCAGGCTCCCTATGACCCCAACGGAAAGCCAGAAAGGTTCTTTTACAACGTGGAGTCATGCGGCTCTCTGCGACCAGAGACCATCGTAATGTCAGCGCTGGCTGTGCTCAAGAAGAAGCTGAGTGATCTGCAGACTCAGCTGAGCCACGAGATCCAGAGCGACGTGCTCACCATCAACTGAGGGACACTCCCCAGACAGAGCAAGACCTGCTTTTTACAGATTTGTACAGACCAGAGGGGCAGATTCAGGATCGCTGTTCAGGTCACACGGCACACATCCAAATATTTGTTGTGGAGTTAGATGTATCCTCCCCGGATACTGGCCATGGATCTGTATGTGTCTGTTATCAAAGGCCAAGTTGTGATTCTTGAGCTCTTATATGAACCAATAAGAGCTGTAAAAGTTATTATATTAAATGGTTAATTATTAGAAATCATGACTCATTATTTTTGTTATTATTATCATTATTATGAAACCTCCATATCCAAAATTACAAATGATTTTCATTTATCTGGACATTTTTTAGATTATTTCAGCACCAGCAGATATTGTAACATCAGCTGTCCAATATAAGGTCCGTGTCTGAAGGGGAGTTTGGCTCAGAATGATACAAATATAGTTTCATTGTCAGCAGTGTAACACTTACACTATTTGATTATTATGTTAAATGTTCCTTTTTAAATAAATCTGTTTGTGTTCT

>Seq7 [organism=Lateolabrax maculatus ] Lateolabrax maculatus (spotted sea bass) Ribosomal protein L7 (RPL7) mRNA, partial cds

CGGTTCTTTTTTTCAGCCAGCTCTTTCTCGGTGGACGCAATGGCGGACGCAGAAAAAAAGGTTCCGGCGGTCCCTGAGAGCCTTTTGAAAAGGCGAAAGGCCTTCGCCACCATGAAGGCCATGCGTCTCAAGAAGATGCTGGCTGAAAAAAAGAACCGCAAAGTGACCAGGAAACTGATCTACAAGAGGGCTGAGCAGTACCACAAGGAGTACAGGCAGATGTACAGGCGTGAAATCCGCCTGGGTCGCACTGCTCGCAAAGTGGGGAACTACTATGTGCCAGCAGAGCCCAAGCTGGCCTTTGTCATCAGGATCAGAGGTATCAACGGTGTCAGCCCCAAGGTCCGCAAGGTTTTGCAGCTTCTCCGTCTGCGCCAGATCTTCAATGGTGTGTTCGTCAGGCTGAACAAGGCTTCAATCAACATGCTCAGGATTGCCGAGCCTTACATCGCTTGGGGATACCCCAACCTGAAGTCTGTGCGCGAGCTGATCTACAAACGTGGCCATGGCAGAATGAGGAAACAGCGCATTGCCCTCACAGACAACGCTCTGGTGGAGAAGGCTCTTGGCAAATATGGCATCATCTGCGTTGAGGACCTCATCCATGAGATTTACACAGTTGGAAAGAACTTCAAGCCTGCCAACAACTTCCTGTGGCCCTTCAAGCTGTCATCCCCCCGCGGTGGTATGAACAAGAAGACCACACACTTTGTGGAGGGAGGCGACGCTGGCAACAGGGAGGATCAGATCAACAGAATGATCAGGAGGATGAACTAA

>Seq8 [organism=Lateolabrax maculatus ] Lateolabrax maculatus (spotted sea bass) >β-Actin (ACTB) mRNA, partial cds

ATGGAAGATGAAATCGCCGCACTGGTTGTTGACAACGGATCCGGTATGTGCAAAGCCGGATTCGCCGGAGACGACGCCCCTCGTGCTGTCTTCCCCTCCATCGTCGGTCGCCCCAGACATCAGGGAGTGATGGTGGGTATGGGCCAGAAGGACAGCTACGTTGGTGATGAAGCCCAGAGCAAGAGAGGTATCCTGACCCTGAAGTACCCCATCGAGCACGGTATTGTGACCAACTGGGATGACATGGAGAAGATCTGGCATCACACCTTCTACAACGAGCTGAGAGTTGCACCTGAGGAGCACCCAGTCCTGCTCACAGAGGCCCCCCTGAACCCCAAAGCCAACAGGGAGAAGATGACCCAGATCATGTTTGAGACCTTCAACACCCCCGCCATGTACGTTGCCATCCAGGCTGTGCTGTCCCTGTATGCCTCTGGTCGTACCACCGGTATCGTCATGGACTCCGGTGATGGTGTGACCCACACAGTGCCCATCTATGAGGGCTACGCCCTGCCCCACGCCATCCTGCGTCTGGACTTGGCCGGTCGCGACCTTACAGACTACCTCATGAAGATCCTGACAGAGCGTGGCTACTCCTTCACCACCACAGCCGAGAGGGAAATCGTGCGTGACATCAAGGAGAAGCTGTGCTATGTCGCCCTGGACTTCGAGCAGGAGATGGGCACCGCTGCCTCCTCCTCATCCCTGGAGAAGAGCTACGAGCTGCCCGACGGACAGGTCATCACCATTGGCAATGAGAGGTTCCGTTGCCCAGAGGCCCTCTTCCAGCCTTCCTTCCTCGGTATGGAGTCCTGCGGAATCCACGAGACCACCTACAACAGCATCATGAAGTGTGATGTCGACATCCGTAAGGACCTGTACGCCAACACAGTGCTGTCTGGAGGTACCACCATGTACCCCGGCATCGCTGACAGGATGCAGAAGGAGATCACAGCCCTGGCCCCATCCACCATGAAGATCAAGATCATTGCCCCACCTGAGCGTAAATACTCTGTCTGGATCGGAGGCTCCATCCTGGCCTCCCTGTCCACCTTCCAGCAGATGTGGATCAGCAAGCAGGAGTACGATGAGTCCGGCCCCTCCATCGTCCACCGCAAATGCTTCTAA

>Seq8 [organism=Lateolabrax maculatus ] Lateolabrax maculatus (spotted sea bass) >18S rRNA(18S) mRNA, partial cds

TACAGGACTCTTTCGAGGCCCTGTAATTGGAATGAGTACACTTTAAATCCTTTAACGAGGATCAATTGGAGGGCAAGTCTGGTGCCAGCAGCCGCGGTAATTCCAGCTCCAATAGCGTATCTTAAAGTTGCTGCAGTTAAAAAGCTCGTAGTTGGATCTCGGGATCGAGCTGACGGTCCGCCGCGAGGCGAGCTACCGTCTGTCCCAGCCCCTGCCTCTCGGCGCCCCCTCGATGCTCTTAGCTGAGTGTCCCGCGGGGTCCGAAGCGTTTACTTTGAAAAAATTAGAGTGTTCAAAGCAGGCCCGGTCGCCTGAATACCGCAGCTAGGAATAATGGAATAGGACTCCGGTTCTATTTTGTGGGTTTTCTTTCTGAACTGGGGCCATGATTAAGAGGGACGGCCGGGGGCATTCGTATTGTGCCGCTAGAGGTGAAATTCTTGGACCGGCGCAAG
